# Predictive Features of Persistent Activity Emergence in Regular Spiking and Intrinsic Bursting Model Neurons

**DOI:** 10.1371/journal.pcbi.1002489

**Published:** 2012-04-26

**Authors:** Kyriaki Sidiropoulou, Panayiota Poirazi

**Affiliations:** Institute of Molecular Biology and Biotechnology (IMBB), Foundation for Research and Technology-Hellas (FORTH), Heraklion, Crete, Greece; University of Freiburg, Germany

## Abstract

Proper functioning of working memory involves the expression of stimulus-selective persistent activity in pyramidal neurons of the prefrontal cortex (PFC), which refers to neural activity that persists for seconds beyond the end of the stimulus. The mechanisms which PFC pyramidal neurons use to discriminate between preferred vs. neutral inputs at the cellular level are largely unknown. Moreover, the presence of pyramidal cell subtypes with different firing patterns, such as regular spiking and intrinsic bursting, raises the question as to what their distinct role might be in persistent firing in the PFC. Here, we use a compartmental modeling approach to search for discriminatory features in the properties of incoming stimuli to a PFC pyramidal neuron and/or its response that signal which of these stimuli will result in persistent activity emergence. Furthermore, we use our modeling approach to study cell-type specific differences in persistent activity properties, via implementing a regular spiking (RS) and an intrinsic bursting (IB) model neuron. We identify synaptic location within the basal dendrites as a feature of stimulus selectivity. Specifically, persistent activity-inducing stimuli consist of activated synapses that are located more distally from the soma compared to non-inducing stimuli, in both model cells. In addition, the action potential (AP) latency and the first few inter-spike-intervals of the neuronal response can be used to reliably detect inducing vs. non-inducing inputs, suggesting a potential mechanism by which downstream neurons can rapidly decode the upcoming emergence of persistent activity. While the two model neurons did not differ in the coding features of persistent activity emergence, the properties of persistent activity, such as the firing pattern and the duration of temporally-restricted persistent activity were distinct. Collectively, our results pinpoint to specific features of the neuronal response to a given stimulus that code for its ability to induce persistent activity and predict differential roles of RS and IB neurons in persistent activity expression.

## Author Summary

Memory, referred to as the ability to retain, store and recall information, represents one of the most fundamental cognitive functions in daily life. A significant feature of memory processes is selectivity to particular events or items that are important to our survival and relevant to specific situations. For long-term memory, the selectivity to a specific stimulus is seen both at the behavioral as well as the cellular level. For working memory, a type of short-term memory involved in decision making and attention processes, stimulus selectivity has been observed *in vivo* using spatial working memory tasks. In addition, persistent activity, which is the cellular correlate of working memory, is also selective to specific stimuli for each neuron, suggesting that each neuron has a ‘memory field’. Our study proposes that both the location of incoming inputs onto the neuronal dendritic tree and specific temporal features of the neuronal response can be used to predict the emergence of persistent activity in two neuron models with different firing patterns, revealing possible mechanisms for generating and propagating stimulus-selectivity in working memory processes. The study also reveals that neurons with different firing patterns may have different roles in persistent activity expression.

## Introduction

Working memory reflects the temporary storage of information that is necessary for immediate decisions/actions. Delay-period activity, which corresponds to neural activity that persists after the end of the initiating stimulus, represents the cellular correlate of working memory [1], [2]. This activity, referred from now on as persistent activity, is stimulus-selective: a specific pyramidal neuron will only exhibit persistent activity if a stimulus is presented in specific locations of the visual field, in the spatial working memory tasks for example, which represents the neuron's memory field. [3], [4]. ‘A large body of work has been devoted to understanding the biophysical mechanisms underlying induction and maintenance of persistent activity, which have emphasized the importance of a delicate balance between excitatory and inhibitory recurrent network connections [5], [6], [7], [8], [9], as well as the contribution of intrinsic cellular conductances [10], [11], [12], [13]. However, very little is known regarding the cellular mechanisms that enable stimulus selectivity in the PFC. How does a neuron ‘recognize’ the relevant stimulus and therefore, enters a persistent activity state? Previous studies have suggested that formation of these memory fields entails proper inhibitory transmission [14], as well as fine interactions between pyramidal neurons and interneurons [15], similar to the mechanisms underlying the formation of orientation columns [16]. However, additional cell-specific features, such as the latency to the first action potential or the sequence of inter-spike intervals (ISIs), could also be involved in the formation of memory fields, as shown in the visual cortex [17].

In the prefrontal cortex (PFC), the brain area heavily involved in mediating working memory functions and expression of persistent activity, layer V pyramidal neurons come in at least two flavors with respect to their firing patterns: intrinsic bursting (IB) neurons, characterized by an initial burst of action potentials (APs) followed by single APs or regular spiking (RS) neurons, characterized by a sequence of single APs [18], [19]. These neurons can also be categorized as adapting, whose firing frequency in response to a constant current step decreases during the stimulation and non-adapting [20], [21]. These different pyramidal neuron subtypes based either on their morphology or firing pattern, can form distinct sub-networks [22], [23], [24] that project to different subcortical areas, such as the pons or the striatum, suggesting that they might serve distinctive functional roles. This is further supported by recent data showing that cortico-pontine pyramidal neurons, compared to cortico-cortical neurons, have increased levels of the hyperpolarization activated cation current (H-current), contributing to increased temporal summation and increased amplitude of the slow afterdepolarization (dADP), which in turn facilitates the probability of persistent activity induction [25].

The present study uses detailed compartmental models of IB and RS neuron sub-types to identify (a) features of incoming signals that determine persistent activity induction (stimulus selectivity) and (b) characteristics of the neuronal response to these signals that may be used by downstream neurons to decode information about the probability of persistent activity emergence (encoding of preferred stimuli). Our results predict that stimulus-selectivity is tightly linked to the spatial location of activated synapses. Moreover, while the properties of persistent activity differ between the two subtype models, in both neurons the latency to the first action potential and the initial inter-spike-intervals of the stimulus-induced response contain predictive information regarding the emergence of persistent activity, providing a mechanism for encoding and propagating the occurrence of preferred signals.

## Results

Following the construction of a morphologically and biophysically-detailed layer V PFC pyramidal neuron model, biophysically relevant variations in the sodium and R-type calcium currents (see Methods) led to the emergence of two distinct neuronal sub-types: a Regular Spiking (RS) and an Intrinsic Bursting (IB) model neuron. Specifically, a combination of doubling the R-type calcium and the persistent sodium conductances changed the firing pattern of the model neuron from an RS to an IB one. The experimentally documented range of these conductances [26], [27] indicates that such differences are often seen in layer V PFC pyramidal neurons.

**Figure 1:**
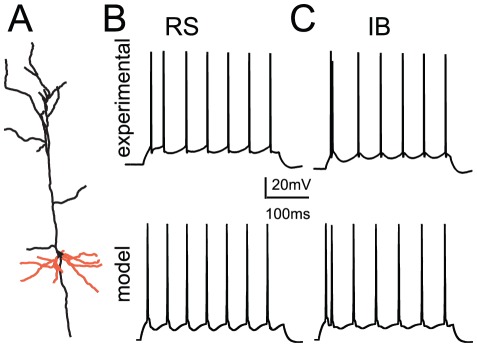
Neuron models used. A: Neuronal morphology used to construct the model neurons. B, C: Experimental traces (top) and voltage responses of the regular spiking (B) the intrinsic bursting (C) model neurons (bottom) in response to a somatic step-pulse current injection (200 pA). Experimental traces taken from the database used in [11].

Neuronal responses were first validated extensively against known experimental data in order to verify that both model neurons exhibit: a) physiological values of input resistance (81 MΩ for both model neurons, experimental average 79.60±6.6 MΩ [28]), b) physiological responses to step pulse current injections (Fig. 1B, C), c) proper back-propagating action potentials (BPAPs) in the apical as well as the basal dendrites (Supplemental Fig. S1) and d) physiological synaptic responses in the basal dendrites (Supplemental Fig. S2, also see Methods).

### Validation of the NMDA and dADP mechanisms

Two biophysical mechanisms have thus far been implicated in the generation of persistent activity: the NMDA [7], [9], [29], [30] and the CAN conductance [7], [10], [11], [29].

**Figure 2:**
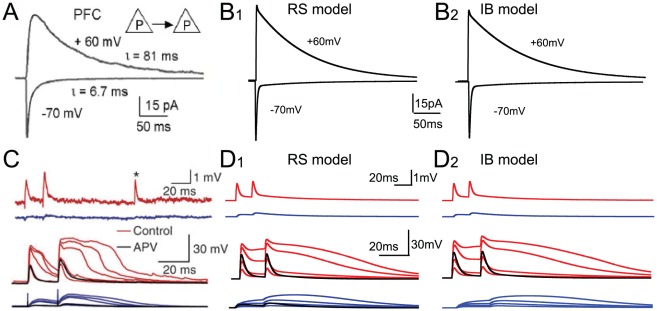
Validation of the NMDA mechanisms. A. Experimental traces showing current responses at the soma of a pyramidal neuron following generation of an action potential in a nearby connected pyramidal neuron within the PFC [31]. The current response at −70 mV corresponds to the AMPA current while the current response at +60 mV corresponds to the NMDA current. B. Simulated somatic current recordings at −70 mV (AMPA) and at +60 mV (NMDA) in response to activation of 5 synapses at the basal dendrites of the RS (B1) and the IB (B2) model neuron. C. Experimental traces showing generation of NMDA spikes at the basal dendrites of cortical neurons [32]. Top two traces correspond to dendritic (red) and somatic (blue) traces in response to unitary glutamate uncaging. The bottom red traces correspond to dendritic voltage responses to increasingly more glutamate uncaging and the bottom blue traces to the corresponding somatic responses. The black trace (amongst the red ones) corresponds to the dendritic voltage response in the presence of NMDA receptor blocker APV. D. Simulated dendritic and somatic voltage responses of the RS (D1) and IB (D2) model neurons. The top two sets of traces are dendritic (red) and somatic (blue) voltage responses to stimulation at the basal dendrites with increasing number of synapses (10, 20, 40 and 50 synapses, shown as traces with increasing amplitude). The bottom two sets of traces are dendritic (red) and somatic (black) voltage responses to stimulation of an increasing number of synapses at the basal dendrites (10, 20, 40 and 50 synapses, shown as traces with increasing amplitude). In both neuron models, at least 40 synapses were required for NMDA spikes to be generated.

The NMDA current was validated with respect to the AMPA current based on experimental data from connected layer V PFC pyramidal neurons showing that the NMDA-to-AMPA ratio is 1.2, and that NMDA currents have relatively slow kinetics of inactivation (Fig. 2A, B1, B2) [31]. Furthermore, it has been shown that basal dendrites of layer V pyramidal neurons exhibit NMDA spikes at their basal dendrites (Fig. 2C) [32], [33]. These NMDA spikes are generated in an all-or-none manner, and once generated, stronger stimuli affect mostly the duration of the NMDA spike, while a slight increase in the amplitude may also be seen [32]. We tested whether NMDA spikes could be evoked at the basal dendrites of the neuron models. A dendritic branch located about 100 µm from the soma was stimulated with an increasing number of excitatory synapses. When using at least 40 excitatory synapses to induce the necessary depolarization, dendritic NMDA spikes could be evoked in both model neurons. Further increase in the number of synapses resulted in an increase of the NMDA spike duration along with a slight increase in the spike amplitude, in accordance with the experimental data (Fig. 2D1, D2).

**Figure 3:**
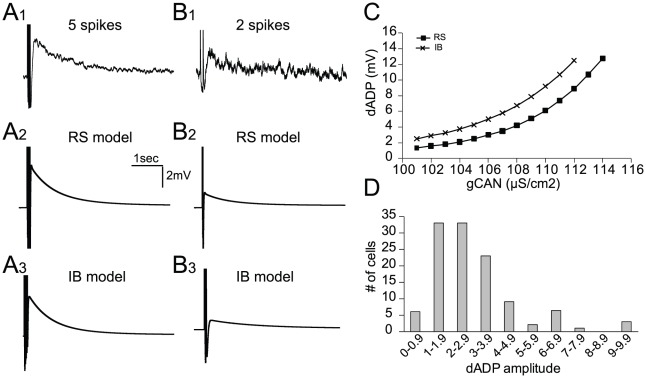
Modeling the dADP following somatic current injection. A, B. *Top traces*. Experimental traces of the dADP in a layer V PFC pyramidal neuron following 5 action potentials (A1) or 2 action potentials (B1) in response to 5 ms step pulses at 20 Hz. *Bottom traces*. Simulated traces of the dADP following activation of the CAN mechanism in the RS model after 5 APs (A2) or 2 APs (B2) and in the IB model after 5 APs (A3) or 2 APs (B3). C. Increasing the conductance of the CAN mechanism in both model neurons (RS and IB) increases the dADP induced following 5 action potentials. D. The distribution of all neurons recorded experimentally based on their dADP. All values of dADP used in simulations fall within this physiological range (taken from [11]).

Layer V PFC pyramidal neurons have been shown to exhibit a delayed afterdepolarization (dADP) following stimulation of G_q_-coupled receptors [34], [35]. This dADP is induced following a burst of action potentials and has small amplitude (average ∼3 mV) and very slow kinetics (decay τ = 3 sec) [35], rendering it a possible mechanism for induction and maintenance of persistent activity [11]. The dADP has been shown to be primarily generated by the CAN current [36] and possibly results from the activation of TRPC4/5 channels which are found in layer V PFC pyramidal neurons [35]. We simulated the dADP by including an additional ionic mechanism, which is mainly dependent on two variables: a) the half point of calcium-induced activation and b) the rate of inactivation. These two variables were adjusted so that the dADP was induced following a burst of 5 spikes, but was much smaller following just 2 spikes (Fig. 3A, B), in accordance with experimental findings [11]. The amplitude of the dADP could be modified by changing the conductance of the CAN mechanism (Fig. 3C) and was kept within the physiological range (2–8 mV), based on existing experimental data ([11] and Fig. 3D).

### Induction of persistent activity in a single neuron model

Cortico-cortical connections that are thought to underlie the emergence and maintenance of persistent activity [7], [37] form synapses onto the basal dendrites of pyramidal neurons [38]. Therefore, the basal dendrites of the model neurons were stimulated with a total of 200 excitatory synapses (containing both AMPA and NMDA receptors), evenly distributed within a few basal dendrites (see Methods), 10 times at 20 Hz (synchronously), while the soma was stimulated with 5 inhibitory synapses (both GABA_A_ and GABA_B_) at 50 Hz (also synchronously) [39]. This stimulation protocol was repeated 50 times and the location (set of dendritic branches), but not the stimulation time, of activated synapses varied between trials (see Methods). Persistent activity was induced in a probabilistic manner, in a percentage of these trials.

Synaptic stimulation alone (in the absence of the CAN mechanism) did not lead to persistent activity in any single cell model, even when the number of stimulated synapses was gradually increased up to 400. However, since neuromodulators, such as dopamine and serotonin, are known to increase NMDA currents in layer V PFC pyramidal neurons [40], [41], [42] and the NMDA conductance is required in large-scale networks for stabilizing persistent activity [7], we next tested whether increasing the NMDA current by 25% could induce persistent activity in the single neuron models. Increasing the NMDA-to-AMPA ratio (abbreviated “N*”, with * equal to the ratio) from 1.2 to 1.5 did not induce persistent activity in any model neuron (data not shown) although it resulted in decreased inter-spike-intervals (ISIs) of the neuronal response during the stimulus (Supplemental Table S1). The latter concurs with experimental data showing modulation of neuronal excitability by NMDA *in vitro* [43].

**Figure 4:**
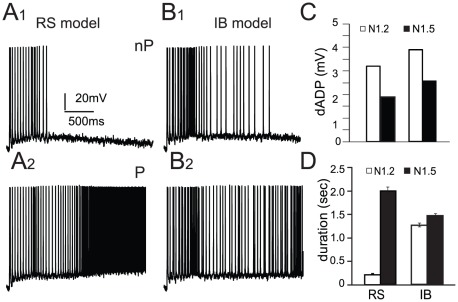
Persistent activity induction was studied in response to a 20 Hz stimulus targeted at the basal dendrites of both model neurons. A–B. Representative traces of a simulation trial exhibiting no persistent activity (top) and a trial exhibiting persistent activity (bottom) for the RS (A) and the IB model neuron (B). C. The magnitude of the dADP required for induction of persistent activity in at least 50% of the trials for the RS and IB neuron models at two different NMDA-to-AMPA ratios, 1.2 (white bars) and 1.5 (black bars) D. Duration of neuronal activity after the end of the stimulus in the no persistent trials for the RS and IB neuron models at two different NMDA-to-AMPA ratios, 1.2 (white bars) and 1.5 (black bars).

Activation of the dADP mechanism on the other hand, resulted in induction of persistent activity, that is, neuronal activity that lasted more than 3 seconds following the end of the stimulus (Fig. 4A2, B2) in both neuron models. Increasing the magnitude of the CAN conductance (*i.e.*, increasing the amplitude of the resulting dADP, tested with five somatic step pulses, within the physiological range) increased the probability of inducing persistent activity. We characterized the magnitude of the CAN current that would induce persistent activity with at least 50% probability in the 50 experimental trials in which the spatial arrangement of the synapses on basal dendrites was varied (*i.e.*, at least 25/50 trials exhibited persistent activity). The dADP required for induction of at least 50% persistent activity for the RS and IB neuron models was 3.2 and 3.9 mV, respectively (Fig. 4C, white bars) and dropped by 1.3 mV in both models when the NMDA-to-AMPA ratio increased to 1.5 (Fig. 4C, black bars). Note, however, that the slightly larger dADP in the IB model cell corresponds to a smaller CAN conductance compared to the RS model cell (Fig. 3C). This can be explained by the enhanced R-type calcium and persistent sodium currents in the IB model cell which may contribute to the long-lasting depolarization produced by the CAN mechanism, thus partially substituting the CAN conductance. Taken together, these findings show that induction of persistent activity requires a larger dADP (although a smaller CAN conductance) in the IB than the RS model cell.

In the previous analysis, we classified persistent activity as the neuronal activity that continues past the end of the initiating stimulus and lasts at least 3 seconds. However, the neuron models could exhibit self-terminated persistent activity (500–2000 ms) (Fig. 4D, white bars), even in the experimental trials classified as not having persistent activity (‘no persistent’ trials). We notice that the RS neuron model exhibits significantly less temporally-restricted persistent activity compared to the IB neuron model (Fig. 4D, white bars, p<0.001). Increasing the NMDA-to-AMPA ratio however facilitates short-lasting persistent activity to a much larger extent in the RS than the IB model (Fig. 4D, black bars, p<0.001). A possible explanation could lie in the fact that the IB model cell has larger R-type calcium and persistent sodium currents, which together with the CAN mechanism contribute to the prolonged depolarization needed for persistent activity. Thus, an additional slow increase in Ca^++^ influx due to enhanced NMDARs would have a greater impact on the RS model, where the primary conductance responsible for the dADP is the CAN conductance, than the IB model, where several mechanisms -with different kinetics- already contribute to this depolarization. Furthermore, this short-lasting persistent activity could be significant in an *in vivo* situation where network mechanisms could maintain it for longer periods of time. These findings show that, while persistent activity in the single neuron models is primarily dependent on the CAN current, altering the NMDA-to-AMPA ratio modulates the duration of persistent activity that lasts less than 3 seconds.

Having characterized the conditions leading to persistent activity emergence in both model cells, our next goal was to search for features of the input and/or the models' response that would be associated with stimulus-selectivity.

### Synaptic location as a feature of stimulus-selectivity

The presence of ‘memory fields’ has been shown in individual PFC neurons with respect to delay-period activity [44]. That is, a specific neuron exhibits robust delay-period activity (*i.e.*, an increase in firing rate during the delay compared to the stimulus period) only for a specific set of locations in the visual field [3]. The way a PFC pyramidal neuron, however, identifies its memory field remains an open question. It is possible that different incoming stimuli, such as stimuli located in different parts of the visual field, activate synapses in different dendritic branches on PFC pyramidal neurons and this spatial specificity of inputs is in turn used to discriminate between preferred (*i.e.*, those leading to persistent activity) and non-preferred stimuli. In our models, we used 50 simulation trials, in which the set of dendritic branches containing synaptic mechanisms varies with each trial (see Methods). This variability in the location of incoming contacts could be assumed to represent different incoming stimuli [45], hence, we conjecture that the spatial location of activated synapses may play a role in persistent activity induction.

**Figure 5:**
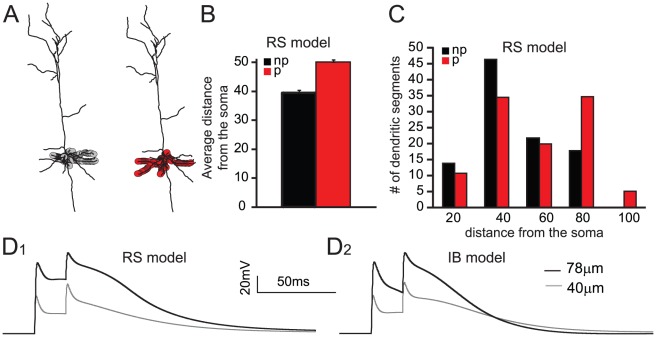
Characteristics of the spatial arrangement of synapses in ‘persistent’ and ‘no persistent’ trials. A. Representative spatial arrangements from a ‘no persistent’ (black) and a ‘persistent’ (red) trial in the RS model neuron, showing that the synapses in a ‘no persistent’ trial are closer to the soma compared to the synapses in the ‘persistent’ trial. B. Graph showing the average distance from the soma of all dendritic branches activated in ‘persistent’ and ‘no persistent’ trails in the RS model neuron. C. Histogram of the average distance from the soma of all activated dendritic branches in the RS model neuron. D. Dendritic voltage traces showing that NMDA spikes are larger when a dendrite located at 78 µm from the soma (black trace) is stimulated with 20 synapses, compared to the NMDA spikes generated in a dendrite located at 40 µm from the soma (grey trace) in the RS (D1) and the IB (D2) model neurons.

To test this hypothesis, we measured the distance from the soma and the center of each dendritic branch that contained stimulated synapses, and averaged the values of each of these features for all dendritic branches in ‘persistent’ versus ‘no persistent’ trials. We found that in both neuron models, synaptic mechanisms were on average located further away from the soma for the ‘persistent’ trials, compared to the ‘no persistent’ trails and this difference was statistically significant (p<0.001) (Fig. 5B, RS model and Supplemental Fig. S3, IB model). The distributions of all activated dendritic segments in ‘persistent’ and ‘no persistent’ trials (Fig. 5C, RS model and Supplemental Fig. S3, IB model) show that this difference stems from a rightward shift as well as a change in the shape of the ‘persistent’ trial distribution due to the activation of dendritic segments located further away from the soma.

A possible mechanistic explanation as to why inputs that are further away from the soma lead to persistent firing may be linked to the generation of NMDA spikes. As shown in Fig. 5D, the magnitude of NMDA spikes is much larger when synapses are stimulated in distal compared to proximal locations within the basal dendrites of both model neurons. It is thus possible that distal inputs lead to persistent activity emergence via the facilitation of NMDA spikes which in turn promote the supralinear integration of synaptic inputs [46] and provide much larger and longer lasting somatic depolarizations. These findings are supported by recent experimental data showing that inputs to proximal basal dendrites of cortical pyramidal neurons sum linearly and require precise temporal coincidence for effective summation, whereas distal inputs are combined supralinearly over broader time windows in an NMDAR-dependent manner [47]. Finally, these findings suggest that the relative distance of incoming signals from the cell body may code for the neuron's memory field and therefore, their ability to induce persistent activity.

### Initial spikes in the stimulus-induced response code for persistent activity emergence

Since the spatial location of incoming contacts is significantly different between ‘persistent’ and ‘no persistent’ trials, it is likely that these differences are reflected in the neuronal response to these stimuli and, if so, this information can be used by downstream neurons to decode the upcoming emergence of persistent activity before it occurs [48].

**Figure 6:**
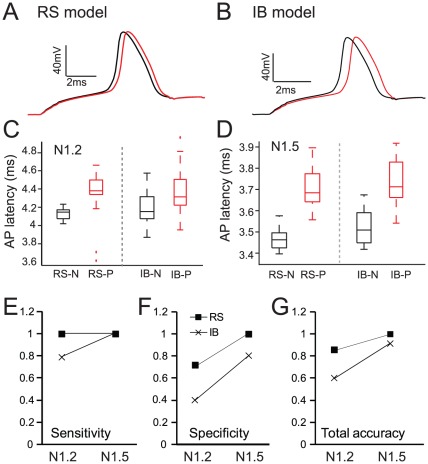
The AP latency of the stimulus-induced response can discriminate between ‘persistent’ and ‘no persistent’ trials. A–B. Representative traces showing the increased AP latency in the ‘persistent’ trials compared to ‘no persistent’ trials in the RS (A) and IB (B) models. C–D. Box plots of the first AP latency in trials where persistent activity is induced (red) and in trials where no persistent activity (black) is induced for the RS and IB neuron models when N = 1.2 (C) and N = 1.5 (D). E–G. Sensitivity (E), Specificity (F) and Total Accuracy (G) obtained when using Linear Discriminant Analysis on the AP latencies of the RS and IB neuron models.

To test this hypothesis, we first examined whether features of the neuronal response to the stimulus, such as the average firing frequency or the AP latency differed between preferred and non preferred inputs. We found that the average ISIs of the neuronal response during the stimulus was not different between ‘persistent’ and ‘no persistent’ trials in either the RS or the IB neuron model (see Supplemental Table S2). However, the first AP latency of the models' response was clearly different in the ‘persistent’ trials when compared to the ‘no persistent’ trials (see Fig. 6A–D and Supplemental Table S3). Specifically, ‘persistent’ trials in the RS model had AP latencies that were significantly larger than the ‘no persistent’ trails for both NMDA-to-AMPA ratios tested (p<0.001 (N1.2) and p<0.001 (N1.5), non-overlapping boxes in Fig. 6C, D). For the IB model neuron, differences in the AP latencies were highly significant only when the NMDA-to-AMPA ratio was increased to N1.5 (p = 0.0065 (N = 1.2), overlapping boxes in Fig. 6C and p<0.001 (N = 1.5), non-overlapping boxes in Fig. 6D). In both models, persistent activity emergence was associated with a slightly slower onset of the neuronal response, which could be explained by the more distal location of activated synapses, compared to the ‘no persistent’ trials (Fig. 5B–C and Supplemental ). Although the differences in the AP latencies between ‘persistent’ and ‘no persistent’ trials were small (200–300 µs), recent studies have shown that even submillisecond differences in AP emergence or width could represent meaningful coding parameters for neurons [49], [50], [51], suggesting that the magnitude of the AP latency maybe used to code for the occurrence of a preferred stimulus.

To test whether differences in the AP latency can discriminate between ‘persistent’ and ‘no persistent’ trials in a more systematic manner, we assessed the ability of the AP latency values to predict the emergence of persistent activity using Linear Discriminant Analysis (LDA). For this, we used a training set (consisting of the AP latencies for 20 ‘persistent’ and 10 ‘no persistent’ trials) in order to determine the optimal cut-off that separates the two distributions based solely on the value of the AP latency. The method was validated using leave-five-out cross validation (LFOCV) and subsequently tested on a previously unseen set of another 30 trials (see Methods), to assess how well can the AP latency of the response to a new input determine whether this input will induce persistent activity or not. Each observation (i.e. trial) of the test set was passed through the 6 ‘trained’ LDA models produced by the LFOCV and a class label was assigned by each model (0 for ‘no persistent’ and 1 for ‘persistent’). All model outputs were then averaged and if the average was 0.5 or higher, then that specific observation was classified as a ‘persistent’ trial, otherwise it was classified as a ‘no persistent’ trial.

Using the percentage of correctly predicted ‘persistent’ (sensitivity) and ‘no persistent’ (specificity) trials to assess the method's performance accuracy, we found that discrimination was more successful in the RS than the IB model neuron. Specifically, for the RS model and an NMDA-to-AMPA ratio of 1.2, the sensitivity of the method was very high (100% or 1), while the specificity was a bit lower (0.7), resulting in total accuracy of 0.85 (Fig. 6E–F, black squares). This means that out of the 30 trials tested, all 20 ‘persistent’ trials and 7/10 of the ‘no persistent’ trials are correctly identified by their respective AP values. For the IB model, the sensitivity value was 0.8, the specificity was only 0.4, and the total accuracy was 0.6, considerably decreased compared to the RS model (Fig. 6E–F, ‘x’ marks). However, for a larger NMDA-to-AMPA ratio (N 1.5), the performance accuracy was high for both models, with the RS cell reaching 100% and the IB cell reaching 90% (Fig. 6G). Taken together, these findings suggest that the AP latency may be used as a discriminatory feature for signaling whether a given stimulus will or will not lead to persistent firing, that the accuracy of this prediction is higher in the RS than the IB model neuron and is strongly dependent on the NMDA contribution.

**Figure 7:**
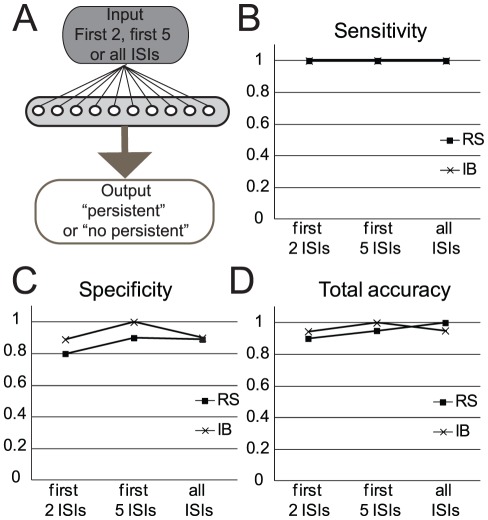
The ISIs of the response to the stimulus can predict the emergence of persistent activity. A. A simple artificial neural network (Perceptron) is used to test whether the ISIs of the stimulus-induced response in the model neurons can predict the emergence of persistent activity. B. The sensitivity of the Perceptron is 100% for both RS and IB model neurons when the first 2, first 5 or all ISIs are used as an input. C. The specificity of the Perceptron for the RS and IB model neurons when the first 2, first 5 or all ISIs are used as an input. D. Total accuracy of the Perceptron for the RS and IB model neurons when the first 2, first 5 or all ISIs are used as an input.

We next investigated whether some other characteristic of the stimulus-induced response can better predict persistent activity emergence even for a lower NMDA-to-AMPA ratio. Towards this goal, we used the ISIs during the stimulus-induced response as input to a linear perceptron (see Methods and Fig. 7A for a graphical illustration) and tested whether ‘persistent’ trials could be discriminated from ‘no persistent’ trials based on these features.

The perceptron was trained and validated with 30 trials (as in LDA), using the leave-one-out cross validation (LOOCV) method and subsequently tested on a previously unseen set of another 30 trials (see Methods and LDA analysis above). Sensitivity and specificity measures were again used to assess the method's performance accuracy. The sensitivity of the perceptron for both the RS and IB neuron models was 100% when the first 2, first 5, or all ISIs of the stimulus-induced response were used as input (Fig. 7B). Similarly, specificity of the perceptron for both models was above 80%, with the IB slightly better than the RS model (0.9 or greater, Fig. 7C) for all ISI sequences tested. In particular, the specificity for the RS model was 80%, 90% and 90% when the first 2, 5 or all ISIs were used as input features (Fig. 7C, black squares) whereas the specificity of the IB model was 90%, 100% and 90%, respectively (Fig. 7C, ‘x’ marks). The perceptron's performance was also assessed on shuffled datasets (in which ‘persistent’ and ‘no persistent’ trials were randomly labeled) and the performance was severely degraded: the sensitivity dropped to 0% and the specificity to 60% for both RS and IB models. These results show that the initial ISIs of the stimulus-induced response contain highly accurate predictive information regarding the emergence of persistent activity in both the IB and the RS model neurons.

Overall, our findings suggest that temporal characteristics of the stimulus-induced response, such as the first spike latency and the first 2 ISIs, contain significant predictive information about the emergence of persistent activity beyond the end of preferred stimuli while average characteristics such as the firing frequency don't capture such information, in accordance with data from other brain regions [48], [52], [53]. These findings are particularly important as they pinpoint specific features of the neuronal response, at the single neuron level, which are common across two major sub-types of pyramidal neurons and which encode stimulus preference with respect to persistent activity emergence. If experimentally validated, these findings suggest a potential mechanism by which stimulus-selectivity that initiates in primary cortices may be decoded by downstream PFC pyramidal neurons within less than 100 miliseconds from the stimulus presentation, and this rapid decoding may have serious implications for the expression of goal-directed behaviors that have been documented in the PFC [54].

### Differential persistent activity properties in RS and IB model cells

Since both model neurons seem to use similar codes for stimulus-selective persistent activity induction, we wondered whether their different firing patterns influenced persistent activity at a different level. We thus contrasted the properties of persistent activity, such as its induction threshold, firing frequency and firing pattern and their dependence on CAN and NMDA in the two model cells.

#### The role of CAN and NMDA conductances

**Figure 8:**
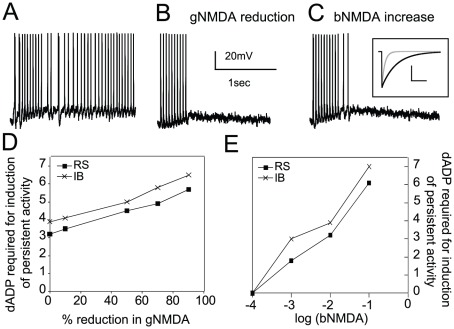
Effect of NMDA current properties on persistent activity induction. A. Stimulus-induced persistent activity in response to the 20 Hz input at the basal dendrites when the NMDA-to-AMPA ratio is 1.2. B. Corruption of stimulus-induced persistent activity when the NMDA conductance is reduced to 90% of its original value. C. Corruption of stimulus-induced persistent activity when the factor for the inactivation time constant of the NMDA conductance is increased 10 times. Inset: NMDA current traces under ‘control’ (black) conditions and under conditions of increased inactivation time constant (grey trace). Scale bars: 1 pA, 50 ms. D. Graph showing the increase in the dADP required for induction of persistent activity while the NMDA conductance is reduced. E. Graph showing the change in dADP required for induction of persistent activity when the factor for inactivation time constant changes (all experiments in this study were performed with a control value of log(bNMDA) = −2).

We first investigated how the biophysical properties (magnitude and kinetics) of NMDA current may contribute to persistent activity induction. We found that a small reduction in the NMDA conductance by 10% (while retaining the same level of excitability by increasing the AMPA conductance) resulted in corruption of persistent activity in both the RS and IB model neurons, (Fig. 8A, B), while a similar decrease of AMPA conductance did not have any effect (data not shown). Only when the dADP was increased could persistent activity be expressed (Fig. 8D). If the NMDA conductance was decreased further, then greater increases in the dADP were required for induction of persistent activity (Fig. 8D). The dADP required for reinstating persistent activity under reduced NMDA conductance conditions was consistently larger in the IB compared to the RS model, similar to the findings of Fig. 4C under control conditions. Furthermore, increasing the inactivation kinetics of the NMDA current (*i.e.*, making the NMDA current faster), by decreasing the inactivation factor 10-fold, resulted in corruption of persistent activity (Fig. 8C) and a substantial increase in the dADP was again required for persistent activity reinstatement (Fig. 8E). On the other hand, a decrease in the inactivation kinetics reduced the dADP required for persistent activity initiation. When the factor for the inactivation time constant matched the one of the CAN mechanism (b = 0.00001), then no dADP was needed for induction of persistent activity in either model neurons (Fig. 8E). This finding suggests that persistent activity induction depends primarily on the long lasting activation of a depolarizing current, which does not necessarily need to be provided by the CAN conductance. If another conductance had similar kinetics, the CAN mechanism could be obsolete. However, since none of the remaining currently known conductances in layer V pyramidal neurons has similar activation/inactivation characteristics, the CAN mechanism remains the key candidate for controlling persistent activity induction.

Collectively, these data show that while the CAN mechanism is necessary to initiate persistent activity in a single model neuron, the NMDA synaptic mechanism -both its conductance and inactivation kinetics- contributes to the emergence of persistent activity and modulates the dADP amplitude required for its induction.

#### Firing characteristics of persistent activity

**Figure 9:**
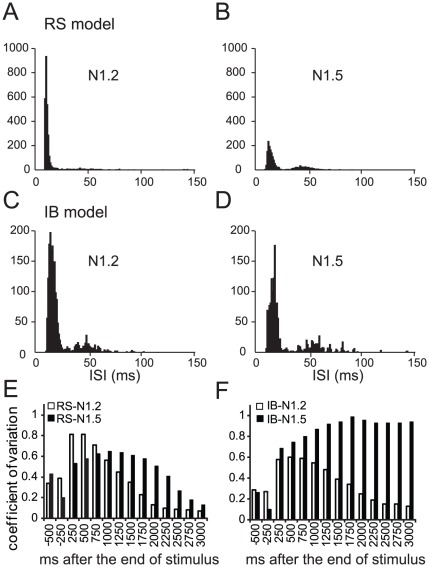
Properties of persistent activity. A, B. Histograms of the ISIs during persistent activity in the RS neuron model for N = 1.2 (A) and N = 1.5 (B). The unimodal distribution seen when N = 1.2 is converted to a bimodal distribution when N = 1.5. C, D. Histograms of the ISIs during persistent activity in the IB neuron model for N = 1.2 (C) and N = 1.5 (D), showing a bimodal distribution under both conditions. E, F: Coefficient-of-variation (CV) before and during persistent activity measured per 250 ms bins. In the RS model neuron (E), CV is initially increased (white bars) or rising (black bars) and gradually decreased during persistent activity when N = 1.2 (white bars) and N = 1.5 (black bars). In the IB model neuron (F), CV gradually decreases during the time course of persistent activity when N = 1.2, but increases and stabilizes to values close to 1 when N = 1.5.

Two important properties of persistent activity are its average firing rate and its coefficient of variation (CV). We find that in both neuron models the average firing frequency increases during persistent activity compared to the stimulus-induced neuronal activity (Supplemental Table S1). Such an increase is observed mostly in neurons that exhibit stimulus-selective persistent activity *in vivo* [55]. In addition, we observe that persistent activity induced in the RS model neuron exhibits a regular firing pattern (Fig. 9A1, unimodal ISI distribution) while persistent activity induced in the IB model neuron exhibits a bursting firing pattern (Fig. 9B1, bi-modal ISI distribution), suggesting that neurons with different stimulus-induced firing pattern responses also exhibit different persistent activity patterns. Increasing the NMDA-to-AMPA ratio allows the RS model neuron to also exhibit bursts during persistent activity, suggesting that the firing pattern of persistent activity could be modulated (Fig. 9A2).

Delay-period activity firing *in vivo* is highly variable, a characteristic that has not been successfully reproduced in existing computational models [55]. We analyzed the variability of the persistent activity generated in our model neurons using the index of coefficient of variation (CV) estimated over 250 ms bins. When the NMDA-to-AMPA ratio is 1.2, CV is initially increased compared to the CV during the stimulus response (see Supplemental Table S1), but gradually decreases to very small values, in both RS and IB model neurons. When the NMDA-to-AMPA ratio increases to 1.5, the CV in the RS model starts out lower, gradually increases and then decreases again during the time course of persistent activity. On the contrary, the CV in the IB model neuron increases gradually over the course of persistent firing and reaches a plateau close to 1 (Fig. 9C). Our results suggest that IB neurons under conditions of increased NMDA receptor contributions can achieve high degrees of variability during persistent activity. In line with the *in vivo* findings [55], the variability *during* persistent activity in both models is similar or larger than the variability during the stimulus-induced response (Supplemental Table S1), despite the increased firing frequency. Given that in our neuron models, persistent activity is maintained primarily by the CAN current (absence of ongoing network input), these results suggest that intrinsic mechanisms, such as the CAN current, cannot explicitly account for the increased variability observed *in vivo*. Increased NMDA contribution from recurrent excitation is likely to further enhance variability in fully connected PFC networks [56].

Overall, we find that the properties of persistent activity such as its firing pattern characteristics, Coefficient of Variation and its dependence on CAN and NMDA currents are differentially expressed in the RS versus the IB model cells, perhaps suggesting a different contribution of these sub-types in working memory.

For example, our models predict that IB model neurons require a larger dADP to induce persistent activity in response to stimulation of their basal dendrites, suggesting that this subtype might be less likely to express persistent activity *in vivo*. On the other hand, stimulus-selective persistent activity properties observed *in vivo* are much closer to the properties of the IB model, suggesting that this subtype is crucial in the proper expression of persistent activity.

## Discussion

In this study, we used a modeling approach to investigate the mechanisms that underlie stimulus-specific induction of persistent activity in two major subtypes of layer V PFC pyramidal neurons: regular spiking and intrinsic bursting cells. We found that persistent activity can emerge in both single neuron models when the basal dendrites are stimulated with realistic synaptic inputs, provided that the CAN mechanism is activated. In addition, we showed that RS and IB model neurons have distinct persistent activity properties, such as different firing patterns during persistent activity as well as differences in its modulation by the NMDA current. More importantly, our findings suggest that the spatial arrangement of activated synapses may determine whether a given signal will lead to persistent activity induction, thus pinpointing a mechanism for stimulus selectivity. Specifically, we found that in both model cells, preferred stimuli consisted of inputs impinging on more distal parts of the basal dendritic tree compared to non-preferred stimuli. Finally, we show that the temporal features of the stimulus-induced response near its onset code for persistent activity induction in both model cells. Specifically, in the RS neuron, both the action potential latency and the first few ISIs are sufficient for discriminating between preferred and non-preferred stimuli while in the IB model neuron only the first few inter-spike-intervals play the same role. These findings suggest the potential decodability of preferred inputs by downstream PFC neurons upon stimulus presentation and long before persistent activity induction.

### Single-neuron persistent activity

The ability of PFC pyramidal neurons to display neuronal activity that persists after the end of stimulation was first recorded *in vivo* in monkeys [57]. This persistent activity has been considered as a network property and particularly a property of recurrent networks due to reverberating excitation [5]. Thus, single neurons have to be part of a recurrent network in order to exhibit persistent activity firing. This hypothesis was further corroborated by the fact that persistent activity could not be induced in neurons recorded in PFC slices where many of the recurrent connections could be severed. Following a modification of the artificial cerebrospinal fluid used, persistent activity lasting for about 1–2 seconds could be recorded from single PFC pyramidal neurons in the slice preparation. This persistent activity, or rather the ‘UP’ state, which occurs both spontaneously and following a stimulus [8], [9], [30], is mediated by AMPA and NMDA [9] and is modulated by GABA_B_ currents [58] and dopamine [59], [60].

Single neurons have been shown to exhibit persistent activity following activation of metabotropic receptors, such as the muscarinic acetylcholine receptor (mAchR) and the metabotropic glutamate receptors (mGluR) [10], [11], [12], due to an underlying depolarizing envelope (*i.e.*, dADP) activated by these receptors [34], [35], [61], [62]. The average dADP in PFC pyramidal neurons following a short 20 Hz stimulus ranges between 2 and 8 mV, not large enough to induce persistent activity by itself, as it has been suggested for enthorhinal cortical neurons [63]. Our computational study shows that this small depolarization when coupled with synaptic activation can induce persistent activity in single neurons.

The common characteristic of both dADP and NMDA mechanisms is their slow inactivation kinetics, previously suggested to be required for the persistent activity to maintain ‘physiological’ firing rates [31]. Our study examined the role of these mechanisms in persistent activity. We showed that while an increased NMDA modulates the neuronal firing rate during the stimulus, increasing the CAN current specifically increases the firing frequency of persistent activity. Furthermore, while increasing the NMDA current increases the variability of firing during persistent activity, increasing the CAN current decreases this variability. Based on analysis from *in vivo* delay-period activity, stimulus-selective -and thus more informative (or significant for mediating behavior)- persistent activity has high firing frequency rates (increased compared to cue-response) as well as increased variability [55]. Our results suggest a dual role for both NMDA and CAN current mechanisms: CAN current acts to enhance persistent activity firing but makes it more regular, while NMDA acts to decrease persistent activity firing but increases its irregularity. Thus, a delicate balance between these two mechanisms *in vivo* is likely to be critical for proper persistent activity firing.

### Synaptic location as a cellular mechanism for stimulus-selectivity

Persistent activity in PFC is stimulus-selective, that is, a neuron will only exhibit persistent firing to specific stimuli, for example stimuli that appear on a specific location of the visual field [44]. The selection of stimuli that a neuron responds to is called a ‘memory field’, in analogy to the receptive fields in the visual cortex [64], or the place fields in the hippocampus [65]. Inhibitory mechanisms play a significant role in shaping the memory fields in PFC, since blockade of GABA_A_ receptors disrupts the emergence of stimulus-selective persistent activity [14].

In our study, we made the assumption that different environmental stimuli could be mapped as different spatial arrangements of synaptic inputs on the basal dendrites. Dendritic activation has been shown to map direction-selective responses in the fly [45] as well as place cells in the hippocampus [66], hence, it is possible that different spatial locations in the neuron's receptive field correspond to the activation of spatially distinct synaptic patterns.

The fact that persistent activity emergence in the model neurons is associated with activation of synapses that are located further away from the soma suggests that perhaps *in vivo* circuits are refined so that stimuli within a memory field project to more distal basal dendrites compared to stimuli outside the neuron's memory field. Since persistent activity is characterized by slow kinetics, it is likely that inputs to distal dendrites, which are generally characterized by slower integration, are more suitable for carrying signals related to persistent activity induction. Finally, stimulation of distal dendrites can generate larger NMDA spikes (Fig. 5 and [67]) which will in turn prolong the window for temporal summation of incoming signals thus resulting in larger and longer-lasting somatic depolarization. Therefore, distal inputs may facilitate persistent activity emergence via the enhancement of NMDA spikes [47].

### Potential decodability of preferred stimuli by downstream circuits

In addition to a strong link between the spatial arrangement of preferred stimuli and the emergence of persistent activity, our data showed that features of the neuronal response during the stimulus such as the AP latency [48] and the first few inter-spike-intervals, can code for the emergence of persistent activity. Specifically, we found that the AP latency in ‘persistent trials’ is on average significantly longer compared to ‘no persistent’ trials in both the RS and IB model neurons. This may be due to the fact that ‘persistent trials’ corresponded to synaptic arrangements in which activated synapses were located significantly further away from the soma than in ‘no persistent’ trials. Although the differences in the AP latencies between ‘persistent’ and ‘no persistent’ trials were submillisecond, several studies suggest that they could still be decoded by downstream neurons [49], [50], [51]. This finding adds to the coding capabilities of the AP latency which has also been found to code for differences in spatiotemporal characteristics of the input in CA1 model neurons [48] as well as the location of sound in secondary auditory neurons [52].

While AP latency was not as powerful predictor at lower NMDA-to-AMPA ratio, particularly in the IB model cell, stimulus selectivity was encoded in the first few inter-spike-intervals of the stimulus-induced response. For both the RS and IB model neurons the emergence (or not) of persistent activity could be predicted with high accuracy when utilizing the first few (2 or 5) ISIs. These findings are particularly important as they suggest the potential decodability of preferred stimuli by neuronal circuits downstream the L5 PFC pyramids, as early as a few hundreds of milliseconds following the stimulus presentation and long before the emergence of persistent activity. In support of this conjecture, recent data suggest that PFC neurons can categorize input signals as early as the stimulus presentation time [68], [69]. This information could in turn be used by downstream striatal [70] and pontine neurons [71], [72] to prepare for the execution of a specific movement and may provide a neuronal basis for goal-directed behavior.

Overall, our findings regarding the coding of information in ISIs are in agreement with studies from other brain regions where ISI sequences were shown to contain more information about receptive fields in the visual cortex than the average firing frequency [17], and could be used to filter and modulate receptive fields in retinal ganglion cells [73]. Recently, *in vivo* patch-clamp techniques uncovered the importance of intrinsic cellular features in active place cell in the hippocampus [74]. Thus, it is now possible to use patch-clamp recordings in PFC pyramidal neurons during virtual working memory tasks to test the prediction that cellular features such as the AP latency or the ISIs can be used to code for the occurrence of preferred stimuli and the emergence of persistent activity.

### Differences between regular spiking and intrinsic bursting neurons

While both IB and RS pyramidal neurons have been documented in the prefrontal cortex [19], [20], their functional role remains unclear. According to recent studies, there could be a link between neuronal sub-types and their preferred target areas. For example, both RS and IB cortical neurons project to the pons (cortico-pontine) or the striatum but no IB neurons project to the contralateral cortex (cortico-cortical) [23]. Similarly, IB neurons in the distal parts of the subiculum project primarily to the medial enthorhinal cortex but not the amygdala [75]. This segregation is likely to be associated with some form of functional specialization of RS and IB neurons. Furthermore, corticopontine neurons, which consist of both RS and IB neurons, in PFC seem more likely to express persistent activity in response to acetylcholine modulation compared to cortico-cortical neurons, in which no IB neurons are found [25]. Our findings are in line with this hypothesis as they support a differential role of RS and IB pyramidal neurons in persistent activity emergence. Moreover, since different neuronal properties have been suggested to provide a recurrent network with different persistent activity characteristics [76], our data suggest that RS and IB neurons may form distinct subnetworks when connected in a recurrent network. Future modeling and experimental work is needed to further investigate this hypothesis.

### Implications regarding the function of dopamine

Dopamine, acting through D1/5 receptors, increases the NMDA current [42] while it decreases the dADP [11]. Our computational study, as well as a previous one [77], suggested that increasing the NMDA component of synaptic stimulation decreases the CAN current required for induction of persistent activity. Thus, D1 signaling seems to modulate both of these mechanisms in order to maintain stability of neuronal excitability. In the case where only NMDA currents are increased while the dADP amplitude in response to metabotropic receptors remains the same, persistent activity will be elicited even in response to non-relevant stimuli. On the other hand, if dADP alone was reduced without any change in the NMDA currents, then no stimulus would be able to induce persistent activity.

Our modeling work showed that when increasing the NMDA current in a similar amount that DA does, the ISI variability increases in both model neurons but it remains elevated for the entire 3 second recoding period of persistent activity only in the IB model neuron (Fig. 9F). Furthermore, increasing the NMDA current also changes the persistent activity properties of the RS model neuron to resemble those of the IB model neuron, with a bursting firing pattern during persistent activity and the emergence of time-limited persistent activity (Fig. 9B and 4D). Our results are in agreement with the well established idea that an increase in DA is necessary for proper expression of persistent activity since the properties of persistent activity in our model neurons are closer to the ones observed *in vivo*, when the NMDA current contribution is increased.

Furthermore, increasing the NMDA current contribution also improves the ‘persistent’ vs. ‘no persistent’ trials discrimination particularly in the IB model neuron, enhancing the coding capabilities of this neuronal subtype, since both the AP latency and/or the first few ISIs can be used to predict the emergence of persistent activity.

DA also modulates other biophysical mechanisms in pyramidal neurons of the prefrontal cortex, such as the L-type calcium channels [78], [79], sodium currents [27], [80] and potassium currents [81], [82]. Modulation of all these mechanisms is likely to affect properties of persistent activity; however, such an analysis is beyond the scope of this work.

### Limitations

Our detailed model reproduces closely the electrophysiological activity of PFC pyramidal neurons. Nonetheless, sources of inaccuracy may have been introduced since the experimental data used to constrain the model are products of *in vitro* preparations. In that sense however, model limitations do not significantly differ from those of the *in vitro* preparations whose findings are readily replicated by the model. Simplifications that have been adopted in this work include: (i) a strictly phenomenogical model of the CAN current, (ii) absence of background synaptic activity which is known to occur *in vivo* (although we do include membrane noise), (iii) stimulation delivered only to the basal dendrites of the model neurons (which are known to receive the majority of inputs from other cortical areas), while more spatially distributed stimulation in combination with the experimentally observed accumulation of extracellular potassium [83] could reduce the threshold for persistent activity induction and require more complex spatial coding features, (iv) same biophysical mechanisms in both model cells, yet different conductance values for the R-type calcium and sodium currents, while in nature, there is probably some variability, and (v) no modeling of plasticity or neuromodulator effects. In spite these simplifications, our model findings are important as they have identified neuronal features that could code for the emergence of persistent activity in both neuronal subytpes found in the cortex, as well as differential properties of persistent activity between RS and IB neurons.

### Concluding remarks

In summary, our modeling results allow the formulation of several predictions, which when tested experimentally could further the current knowledge on persistent activity, its underlying mechanisms and its contribution to working memory. First, we predict that the location of activated synapses is critical for the emergence of persistent firing: stimuli that lead to persistent activity consist of inputs that arrive in distal parts of the basal tree, far away from the soma. This prediction could be easily tested experimentally in slice preparations that generate Up and Down states. Specifically, synaptic stimulation of proximal versus distal basal dendrites should have a different impact on the probability of generating Up states and/or modulating the firing frequency or duration of the Up state. Second, we have identified the AP latency and first few ISIs of the stimulus-induced response as features that could discriminate between stimuli that result in persistent activity or not. These findings could be tested experimentally in slice preparations and/or *in vivo* when a stimulus is used to induce persistent activity. Finally, our models predict that the dADP threshold for persistent activity induction is lower in RS than IB neurons, suggesting that RS neurons should comprise the majority of layer 5 PFC pyramidal neurons that exhibit persistent firing. This can also be tested experimentally in slice preparations that generate Up and Down states, where the effect of synaptic stimulation on the UP state is contrasted between RS and IB neurons. Overall, our modeling work identifies key features of the neuronal response that could predict the emergence of persistent activity and pinpoints a differential role of RS and IB model neurons in persistent activity properties.

## Methods

A detailed compartmental model of a layer V PFC pyramidal neuron comprising of a large variety of membrane mechanisms was implemented in the NEURON simulation environment [84] and was applied on a reconstructed layer V PFC pyramidal neuron available at the neuromorph database (neuron C3_5 from the Smith lab, http://neuromorpho.org/neuroMorpho/index.jsp, shown in Fig. 1A). This neuron was taken from adult Long-Evans rats, at 64–78 days of age [85]. When converted to NEURON, C3_5 had a total of 45 compartments (1 somatic, 1 axonic, 18 basal, and 25 apical dendritic compartments). We assumed a uniform membrane resistance of Rm = 30 kΩ.cm^2^; a uniform intracellular resistivity Ra = 100 Ω.cm; and a specific membrane capacitance of 1.2 µF.cm^−2^ in the soma and 2.0 µF.cm^−2^ in the dendrites. The resting membrane potential was set at −66 mV. Active mechanisms included two types of Hodgkin–Huxley-type Na^+^ currents (transient: I_Naf_; persistent I_Nap;_), three voltage-dependent K^+^ currents (I_Kdr_; I_A_; I_D_), a fast Ca^++^ and voltage-dependent K^+^ current, I_fAHP_; a slow Ca^++^-dependent K^+^ current, I_sAHP_; a hyperpolarization-activated non-specific cation current (I_h_); a low-voltage activated calcium current I_caT_; three types of Ca^++^- and voltage-dependent calcium currents (I_caN_; I_caR_; I_caL_); and the calcium-activated non-selective cation (CAN) current. Channel equations for all the different voltage-gated calcium currents (I_caN_; I_caR_; I_caL_, I_CaT_), I_sAHP_, I_A_ and I_h_ are described in [86], while the channel equations for I_Kdr_, I_D_, I_fAHP_, I_Nap_ and I_Naf_ are described in [56]. A combination of variations of the R-type Ca^++^ current and the persistent Na^+^ current mechanism could generate two different firing patterns: a) a regular spiking (RS) and b) an intrinsic bursting (IB) firing pattern. Specifically, doubling the conductance values for both of the two aforementioned currents switched the firing behaviour of the model neuron from an RS one to an IB one (see Supplementary Text S1). The possibility that such a variability exists in PFC pyramidal neurons is evident from data showing that the current density of persistent sodium current ranges from 2 to 6 pA/pF in PFC pyramidal neurons [27], and that the R-type calcium current contribution could range from 5–25% of total calcium current in layer V pyramidal neurons [26].

The equation for the Ca^++^-activated non-selective cation (CAN) current mechanism was based on [87]. The ‘cac’ and ‘beta’ parameters were adapted so that the current reached its maximum value by the first second following the end of the inducing stimulus and decayed with a time constant of 3 seconds, as observed experimentally (Fig. 3). The actual equations used for the CAN mechanism are the following: 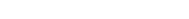, and 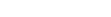, based on [35], where about 70% of the dADP is Na+ current. The state m was calculated by the following set of equations
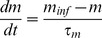
(1)

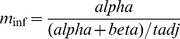
(2)

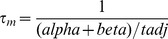
(3)

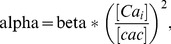
(4)where beta = 0.00001(1/ms) and cac = 0.0004 (mM). The beta and cac values were adjusted so that the dADP was induced following more than 4 spikes and had decay kinetics in the order of a few (∼3) sec.

Furthermore, mechanisms modelling four synaptic currents, AMPA, NMDA, GABA_A_ and GABA_B_, were used in the model neuron (channel equations also described in [86] and Supplementary Text S1). The ‘default’ NMDA-to-AMPA ratio was set to 1.2, based on [31]. The ratio was measured based on the somatic current recorded at −70 mV and +60 mV, following stimulation of 10 synapses on the basal dendrites. We also performed these experiments with the same number of synapses used in the ‘persistent activity’ simulations, and did not find any difference in the ratio of +60 mV and −70 mV currents. In order to achieve this NMDA-to-AMPA ratio, the gNMDA was equal to 3× the gAMPA. In order to increase the ratio to 1.5, the gNMDA was equal to 4× the gAMPA.

### Model validation

Both the RS and IB models were validated with respect to passive and active membrane properties as well as apical and basal dendritic responses (see Supplemental Fig. S1). Dendritic and somatic voltage traces in response to glutamate release were validated based on experimental data [32] (see Supplemental Fig. S2).

### Stimulation protocol

Dendrites were stimulated with a total of 200 excitatory synapses (containing both AMPA and NMDA receptors), equally distributed in 10 different dendritic branches (20 synapses on each branch) which were activated 10 times at 20 Hz. The 10 dendritic branches were selected randomly from the pool of all basal dendrites. Synapses were distributed at random locations within each branch, according to a uniform distribution. The soma of the neuron model was stimulated with 5 inhibitory synapses [39] at 50 Hz. Both excitatory and inhibitory synapses were activated synchronously, without any temporal variability between different trials. Since we were interested in studying the suprathreshold response of neurons to a specific stimulus, we used the above number of synapses for which the neuron model responded with at least 5 APs in the 10 event stimulus.

In addition, for best simulation of membrane potential fluctuations as observed in vitro due to the stochastic ion channel noise [88], [89], an artificial current with Poisson characteristics was injected in the soma of both RS and IB neuron models. This simulation of channel noise is simple compared to ones recently reported [89], yet sufficient for the complexity of the model used and the purpose of the current study.

### Persistent activity

We define persistent activity as the prolongation of neuronal activity following the end of the stimulus for at least 3 seconds. All simulations were recorded for 5 seconds, and if neuronal activity persisted past the 3 seconds following the stimulus, it did not stop before the 5 sec recording. Simulations included 50 repetition trials for each condition (i.e. specific set of NMDA and CAN conductances), where the spatial distribution of activated synaptic mechanisms at the different basal dendritic branches changed in each trial. For the data analysis, only conditions in which persistent activity emerged in at least 50% of the runs were used, unless otherwise noted.

### Data analysis

Estimation of inter-spike-intervals (ISIs) of the simulated neuronal responses, as well as generation of the ISI histograms was performed with custom-made macros using IgorPro software (Wavemetrics, Inc) and Matlab (Mathworks, Inc). Prediction of persistent activity emergence based on the ISI values was done using a custom-made Artificial Neural Network written in Java. The network used was a simple perceptron, which was initially trained with 30 randomly selected trials, validated (using leave-one-out cross validation) and subsequently tested on another 30 trials (both training and test sets comprised of 20 persistent and 10 no persistent trials). Prediction of persistent activity emergence based on the AP latency values was done using Linear Discriminant analysis (LDA) in Matlab (Mathworks, Inc.), with code downloaded from the file exchange site (http://www.mathworks.com/matlabcentral/fileexchange/29673-lda-linear-discriminant-analysis). The method was initially trained with 30 randomly selected trials, validated (using leave-five-out cross validation), and then tested on another set of unseen 30 trials, similarly to the Perceptron analysis. The following conditions were used for the prediction analysis: RS neuron model −N1.2/−N1.5, 40 persistent trials, 20 no persistent trials; IB neuron model −N1.2/−N1.5, 40 persistent trials, 20 no persistent trials (as shown below in the text: N = NMDA-to-AMPA ratio, dADP = delayed afterdepolarization).

### Spatial arrangement analysis

In each trial, synaptic mechanisms were placed according to a uniform distribution within 10 dendritic branches which were selected at random. For each of the selected dendritic branches, the path distance from the center of the branch to the soma was calculated and used to estimate the average dendritic distance for any given trial.

### Experimental results

All experimental results shown here are parts of the neuronal database recorded by Kyriaki Sidiropoulou while she was at Dr. Francis White laboratory and have been reported in previous publications [11], [28].

### Model availability

The model is available for download from ModelDB (Link: https://senselab.med.yale.edu/modeldb/ShowModel.asp?model=144089, accession number: 144089).

## Supporting Information

Figure S1 Validation of dendritic responses in the two neuron models. A–C: Validation of BPAPs in the apical dendrites based on Gulledge and Stuart, 2003 (A1, B1, C1: experimental, A2, B2, C2: simulated, RS model). A1,2: Somatic and dendritic (at 300 µm) traces in response to a 600 ms current step pulse. B1,2: Graphs showing the latency of BPAP in relation to the distance from the soma. C1,2: Graphs showing the relative amplitude of the BPAP (normalized to the somatic BPAP) in relation to the distance from the soma. D–F. Validation of BPAPs in the basal dendrites based on Nevian et al, 2007 (D1, E1, F1: experimental, D2, E2, F2: simulated, IB model). D1,2: Somatic and dendritic (at 300 µm) traces in response to a 500 ms current step –pulse. E1,2: Graphs showing the latency of BPAP in relation to distance from the soma. F1–2: Graphs showing the relative amplitude of the BPAP (normalized to the somatic BPAP) in relation to distance from the soma.
(EPS)

Figure S2 Validation of synaptic responses at the basal dendrites. Experimental (A,B) and simulated (C,D) neuronal responses at the soma (A,C) and dendrite (B, D). For the experimental traces, dendritic synapses were activated with photostimulation of caged glutamate at the dendritic site (Nevian et al, 2007). For the simulated traces, a single AMPA synapse was activated at the dendrite (either proximal or distal). For the simulated responses the red traces correspond to responses following synaptic activation at a distal dendrite (120 µm), and the blue trace corresponds to the responses following synaptic simulation at a proximal dendrite (around 40 µm).
(EPS)

Figure S3 Characteristics of the spatial arrangement of synapses in ‘persistent’ and ‘no persistent’ trials in the IB model neuron. A. Graph showing the average distance from the soma of all dendritic branches activated in ‘persistent’ and ‘no persistent’ runs in the IB model neuron. B. Histogram of the average distance from the soma of all activated dendritic branches in the IB model neuron.
(EPS)

Table S1 Average ISIs (ms) and coefficient of variations during the stimulus and persistent activity.
(PDF)

Table S2 Average inter-spike-intervals (ms) of stimulus-induced activity in trials with or without persistent activity.
(PDF)

Table S3 Action potential latency (ms) in trials with or without persistent activity.
(PDF)

Text S1 Detailed description of the model neurons.
(DOC)
