# Supplementary material for: Predictive Features of Persistent Activity Emergence in Regular Spiking and Intrinsic Bursting Model Neurons
Source: PLoS Comput Biol. 2012 Apr 26;8(4):e1002489. doi: 10.1371/journal.pcbi.1002489 (PMC3343116; doi:10.1371/journal.pcbi.1002489)
Supplement: Table S1 — Average ISIs (ms) and coefficient of variations during the stimulus and persistent activity. (PDF) [file pcbi.1002489.s004.pdf]

Table S1: Average ISIs (ms) and coefficient of variations during the stimulus and persistent activity

	RS neuron model		IB neuron model	
	NMDA-AMPA=1.2	NMDA-AMPA=1.5	NMDA-AMPA=1.2	NMDA-AMPA=1.5
**Stimulus**				
avg ISI	34.4±3.5	19.9±1.8	39.7±4.1	24.3±1.5
cv	0.40	0.44	0.36	0.43
**Persistent**				
avg ISI	14.4±0.6	22.9±1	20.7±0.8	26±0.3
cv	0.77	0.63	0.56	0.82

(*ISI:* interspike stimulus, cv: coefficient of variation)
