# Supplementary material for: Predictive Features of Persistent Activity Emergence in Regular Spiking and Intrinsic Bursting Model Neurons
Source: PLoS Comput Biol. 2012 Apr 26;8(4):e1002489. doi: 10.1371/journal.pcbi.1002489 (PMC3343116; doi:10.1371/journal.pcbi.1002489)
Supplement: Table S2 — Average inter-spike-intervals (ms) of stimulus-induced activity in trials with or without persistent activity. (PDF) [file pcbi.1002489.s005.pdf]

Table S2: Average interspike intervals (ms) of stimulus-induced activity in trials with or without persistent activity

	RS neuron model		IB neuron model	
NMDA-AMPA ratio	‘persistent’ runs	‘no persistent’ runs	‘persistent’ runs	‘no persistent’ runs
1.2	34.3±3.54	38.6±4.53	24.2±2.1	25.6±2.7
1.5	19.9±1.8	21.1±1.9	14.1±0.2	15.7±0.5
