# Supplementary material for: Predictive Features of Persistent Activity Emergence in Regular Spiking and Intrinsic Bursting Model Neurons
Source: PLoS Comput Biol. 2012 Apr 26;8(4):e1002489. doi: 10.1371/journal.pcbi.1002489 (PMC3343116; doi:10.1371/journal.pcbi.1002489)
Supplement: Table S3 — Action potential latency (ms) in trials with or without persistent activity. (PDF) [file pcbi.1002489.s006.pdf]

Table S3: Action potential latency (ms) in trials with or without persistent activity

	RS neuron model		IB neuron model	
NMDA-AMPA ratio	'persistent' runs	'no persistent' runs	'persistent' runs	'no persistent' runs
1.2	4.38±0.03	4.11±0.02	4.4±0.02	4.1±0.02
1.5	3.71±0.02	3.49±0.02	3.70±0.02	3.44±0.02
