# Supplementary material for: Predictive Features of Persistent Activity Emergence in Regular Spiking and Intrinsic Bursting Model Neurons
Source: PLoS Comput Biol. 2012 Apr 26;8(4):e1002489. doi: 10.1371/journal.pcbi.1002489 (PMC3343116; doi:10.1371/journal.pcbi.1002489)
Supplement: Text S1 — Detailed description of the model neurons. (DOC) [file pcbi.1002489.s007.doc]

Supporting Text

1. Distributions of biophysical mechanisms in the model neurons
2. Synaptic mechanisms validation
3. Equations for all biophysical mechanisms used
4. Table showing conductances and distribution of active and passive mechanisms in the model neuron.
5. References
6. **Distributions of biophysical mechanisms in the model neurons**

All the biophysical mechanisms were differentially distributed along the somatodendritic axis, based on available experimental data.

1. Hodgkin–Huxley-type Na^+^ currents (transient: I_Naf_; persistent I_Nap_;)

The conductance of I_Naf_ was highest in the axon, and increased in the soma and proximal dendrites compared to distal and basal dendrite (Gonzalez-Burgos and Barrionuevo, 2001).

1. three voltage-dependent K^+^ currents ( I_Kdr_; I_A_; I_D_).

The conductance of all three different K^+^ currents was decreased in the apical dendrites compared to the soma (Korngreen and Sakmann, 2000; Schaefer et al., 2007)

1. a fast Ca^++^ and voltage-dependent K^+^ current, I_fAHP_; a slow Ca^++^-dependent K^+^ current, I_sAHP_ (Lorenzon and Foehring, 1992), which was present in the soma and much less in the apical dendrites
2. a hyperpolarization-activated non-specific cation current (I_h_), whose conductance was increased, compared to the soma, sigmoidally with a maximum value of 10x that in the soma (Day et al., 2005; Kole et al., 2006) but not the basal dendrites (Nevian et al., 2007)
3. a low-voltage activated calcium current I_caT_ (de la and Geijo-Barrientos, 1996)
4. four types of Ca^++^- and voltage-dependent calcium currents (I_caN_; I_caR_; I_caL_) (Lorenzon and Foehring, 1995)
5. and the calcium-activated non-selective cation (CAN) current responsible for the delayed afterdepolarization (dADP current) (Haj-Dahmane and Andrade, 1998; Fowler et al., 2007). The CAN current was also present in the dendrites but decreased by a factor of 10 (Hagenston et al., 2008)
6. **Synaptic mechanisms validation**

We used experimentally reported data in order to validate the synaptic currents used in our network. The conductance for the AMPA current in the pyramidal cell was validated so as stimulation in the basal dendrite would result to a somatic voltage response of the order of 0.1 mV (Fig. S2A), as reported in (Nevian et al., 2007). The iNMDA to iAMPA ratio at the basal dendrite used in all conditions unless otherwise noted was 1, in accordance with the experimental data of (Wang et al., 2008). This iNMDA conductance could produce NMDA spikes following stimulation of the basal dendrites with 2 events at 50Hz. The conductance of the GABA_A_ mechanism was set so that activation of 1 synapse would result in current amplitude of 10pA, as reported in (Woo et al., 2007).

1. **Equations for all biophysical mechanisms used.**

**Description of synaptic mechanisms.**

***The NMDA receptor (Poirazi et al., 2003)***

$I=g\cdot(V-E_{rev})$ (4)

$g=(R_{on}+R_{off)}\cdot1(ohm^{-1})\cdot B$ (5)

$B=B(V)=\frac{1}{1+\exp(0.072(mV^{-1})\cdot(-V))\cdot(Mg/3.57(mM))}$ (6)

$\frac{dR_{on}}{dt}=\frac{synon\cdot R_{\inf}-R_{on}}{R_{\tau}}$ (7)

$\frac{dR_{off}}{dt}=-\beta\cdot R_{off}$ (8)

$R_{\inf}=\frac{C_{\max}\cdot\alpha}{C_{\max}\cdot\alpha+\beta}$ (9)

$R_{\tau}=\frac{1}{C_{\max}\cdot\alpha+\beta}$ (10)

Where,$E_{rev}=0(mV),Mg=1(mM),C_{\max}=1(mM),\alpha=4(ms^{-1}mM^{-1}),\beta=0.01(ms^{-1})$

***The AMPA receptor (Destexhe et al., 1997)***

$I=g\cdot(V-E_{rev})$ (11)

$g=(R_{on}+R_{off})$ (12) $\frac{dR_{on}}{dt}=\frac{synon\cdot R_{\inf}-R_{on}}{R_{\tau}}$ (13)

$\frac{dR_{off}}{dt}=-\beta\cdot R_{off}$ (14)

$R_{\inf}=\frac{C_{\max}\cdot\alpha}{C_{\max}\cdot\alpha+\beta}$ (15)

$R_{\tau}=\frac{1}{C_{\max}\cdot\alpha+\beta}$ (16)

where $E_{rev}=0(mV),C_{\max}=1(mM),\alpha=10(ms^{-1}),\beta=0.05(ms^{-1})$

***The GABA_A_ receptor (Destexhe et al., 1997)***

$I_{GABA_{A}}=g*(V-E_{rev})$ (17)

$g=(R_{on}+R_{off})$ (18)

$\frac{dR_{on}}{dt}=\frac{(R_{inf}-R_{on})}{R_{\tau}}$ (19)

$\frac{dR_{off}}{dt}=-\beta\cdot R_{off}$ (20)

$R_{\inf}=\frac{C_{\max}\cdot\alpha}{C_{\max}\cdot\alpha+\beta}$ (21)

$R_{\tau}=\frac{1}{C_{\max}\cdot\alpha+\beta}$ (22)

where$E_{rev}=-80(mV),C_{\max}=1(mM),\alpha=5(ms^{-1}mM),\beta=0.18(ms^{-1})$

***The GABA_B_ receptor (Destexhe and Sejnowski, 1995; Pissadaki and Poirazi, 2007)***

$I_{GABA_{A}}=g*(V-E_{rev})$ (23)

$g=(R_{on}+R_{off})$ (24)

$\frac{dR_{on}}{dt}=\frac{(R_{inf}-R_{on})}{R_{\tau}}$ (25)

$\frac{dR_{off}}{dt}=-\beta\cdot R_{off}$ (26)

$R_{\inf}=\frac{C_{\max}\cdot\alpha}{C_{\max}\cdot\alpha+\beta}$ (27)

$R_{\tau}=\frac{1}{C_{\max}\cdot\alpha+\beta}$ (28)

where$E_{rev}=-80(mV),C_{\max}=10(mM),C_{dur}=10(ms),\alpha=0.001(ms^{-1}mM),\beta=0.0047(ms^{-1})$

**Description of ionic mechanisms**

***The leak current***

$I_{l}=g_{l}\cdot(V-E_{l})$, where *E_l_* = *-65 mV* (29)

***The fast sodium channel$I_{Na(fast)}$ (Durstewitz and Gabriel, 2007)***

$I_{Na(fast)}=\overline{g}_{Na(fast)}\cdot(V-E_{Na})$ , where$E_{Na}$ = 55 $(mV)$ (30)

$g_{Na(fast)}=g\max_{Na(fast)}\cdot m\cdot m\cdot m\cdot h$ (31)

$m=\frac{m_{inf}-m}{t_{m}}$ (32)

$m_{inf}=\frac{m_{a}}{m_{a}+m_{b}}$ (33)

$t_{m}=\frac{1}{m_{a}+m_{b}}$ (34)

$m_{a}=\frac{-0.2816\cdot(V+28)}{-1+exp(-(V+28)/9.3)}$ (35)

$m_{b}=\frac{0.2464\cdot(V+1)}{-1+exp(-(V+1)/6)}$ (36)

$h=\frac{h_{inf}-h}{t_{h}}$ (37)

$h_{inf}=\frac{h_{a}}{h_{a}+h_{b}}$ (38)

$t_{h}=\frac{1}{h_{a}+h_{b}}$ (39)

$h_{a}=\frac{0.098}{exp((V+23.1)/20)}$ (40)

$h_{b}=\frac{1.4}{1+exp(-(V+25)/10)}$ (41)

***The persistent sodium channel$I_{Nap}$ (Durstewitz and Gabriel, 2007)***

$I_{Nap}=\overline{g}_{Nap}\cdot(V-E_{Na})$ , where$E_{Na}$ = 55 $(mV)$ (42)

$g_{Nap}=g_{Nap}\cdot m\cdot h$ (43)

$m=\frac{m_{inf}-m}{t_{m}}$ (44)

$m_{inf}=\frac{m_{a}}{m_{a}+m_{b}}$ (45)

$t_{m}=\frac{1}{m_{a}+m_{b}}$ (46)

$m_{a}=\frac{-0.2816\cdot(V+12)}{-1+exp(-(V+12)/9.3)}$ (47)

$m_{b}=\frac{0.2464\cdot(V-15)}{-1+exp(-(V-15)/6)}$ (48)

$h=\frac{h_{inf}-h}{t_{h}}$ (49)

$h_{inf}=\frac{h_{a}}{h_{a}+h_{b}}$ (50)

$t_{h}=\frac{1}{h_{a}+h_{b}}$ (51)

$h_{a}=\frac{0.000028}{exp(-(V+42.8477)/4.0248)}$ (52)

$h_{b}=\frac{0.02}{1+exp(-(V-413.9284)/1488.2589)}$ (53)

***The delayed rectifier potassium current (Durstewitz and Gabriel, 2007)***

$I_{Kdr}=g_{Kdr}*(V-E_{K})$ (54)

$g_{K}=\bar{g}_{Kdr}\cdot n\cdot n\cdot n\cdot n$ (55)

$n=\frac{n_{inf}-n}{t}$ (56)

$n_{inf}=\frac{n_{a}}{n_{a}+n_{b}}$ (57)

$t=\frac{1}{n_{a}+n_{b}}$ (58)

$n_{a}=\frac{-0.018\cdot(V-13)}{-1+exp(-(V+13)/25)}$ (59)

$n_{b}=\frac{0.0054\cdot(V-23)}{-1+exp(-(V-23)/12)}$ (60)

***The fast inactivating potassium current $I_{A}$(Poirazi et al, 2003)***

$I_{Kdr}=\bar{g}_{Ka}\cdot n\cdot l\cdot(V-E_{K})$ (61) $n=\frac{n_{inf}-n}{tn}$ (62) $l=\frac{l_{inf}-l}{tl}$ (63)

$n_{inf}=\frac{1}{1+a}$ (64)

$tn=\frac{betn(v)}{qt+a0n*(1+a)}$ (65)

$l_{inf}=\frac{1}{1+a}$ (66)

***Calcium currents***

***L-type calcium current $I_{Ca(L)}$ (Poirazi et al., 2003; Liebmann et al., 2008)***

$I_{Ca(L)}=(\overline{g}_{Ca(L)init}*(m^{2}\cdot h^{2}+s^{S}*8)*(V-E_{Ca})$ (67)

$\frac{dm}{d(t)}=\frac{m_{inf}-m(t,V)}{\tau_{m}}$ (68)

$\frac{ds}{d(t)}=\frac{s_{inf}-s(t,V)}{\tau_{m}}$ (69)

$m_{inf}=\frac{1}{1+a}$ (70)

$a=alp(V)$ (71)

$alp(V)=exp(\frac{1.e-3*zeta*(V-V_{half})*9.648e4}{(8.315*(273.16+celsius))})$ (72)

where $t_{0}$ = 1.5 $(ms)$, V_half_ = -1 mv, $k_{i}$= 0.025 $(mM)$ the initial calcium concentration.

The current inactivates according to the state *s*, where *S=2*.

$\frac{ds}{dt}=\frac{(s_{inf}-s)}{\tau_{m}}$ (73)

$s_{inf}=\frac{alpha}{alpha+1}$ (74)

$alpha=(\frac{[Ca^{2+}]_{i}}{b})^{2}$ (75)

$\tau_{m}=taumin+\frac{1(ms)*1(mM)}{([Ca^{2+}]_{i}+b)}$ (76)

where b = 0.03 $(mM)$ and $taumin$ = 180 $(ms)$.

***N-type Calcium current (Poirazi et al., 2003)***

$I_{Ca(N)}=g_{Ca(N)_{init}}\cdot m^{2}\cdot h^{3}\cdot([Ca]i)\cdot(V-E_{Ca})$ (89)

$\frac{dm}{d(t)}=\frac{m_{inf}-m(t,V)}{\tau_{m}}$ (90)

$\frac{dh}{d(t)}=\frac{h_{inf}-h(t,V)}{\tau_{h}}$ (91)

$\frac{ds}{d(t)}=\frac{s_{inf}-s(t,V)}{\tau_{s}}$ (92)

$m_{\inf}=\frac{1}{1+a}$ (93)

$a_{m}=a_{m}(V)=\exp(1.e-3\cdot z_{m}\cdot(V-V_{half_{m}})\cdot9.648e4/(8.315\cdot(273.16\_celcius)))$ (94)

$h_{\inf}=\frac{1}{1+b}$ (95)

$b=a_{h}(V)=\exp(1.e-3\cdot z_{h}\cdot(V-V_{half_{h}})\cdot9.648e4/(8.315\cdot(273.16\_celcius)))$ (96)

$s_{\inf}=\frac{a_{2}}{a_{2}+1}$ (97)

$a_{2}=(\frac{k}{[Ca_{i}]})^{2}$ (98)

where $z_{m}=-3.4,\text{ }z_{h}=2,\text{ V}_{\text{half}_{\text{m}}}=-21\text{ (mV), V}_{\text{half}_{\text{h}}}=-40\text{ (mV), k}=\text{0.025 (mM)}$

***R-type calcium current (Poirazi et al., 2003)***

$I_{CaR}=g_{CaR_{(init)}}\cdot m^{3}\cdot h\cdot(V-E_{Ca_{R}})$ (99)

$\frac{dm}{d(t)}=\frac{m_{inf}-m(t,V)}{\tau_{m}}$ (100)

$\frac{dh}{d(t)}=\frac{h_{inf}-h(t,V)}{\tau_{h}}$ (101)

$E_{Ca_{R}}=(1e3)\cdot(R\cdot(celcius\_273.15))/(2\cdot Faraday)\cdot\log([Ca_{o}]/[Ca_{i}])$ (102)

$m_{\inf}=\frac{1}{1+\exp((V+48.5)/(-3))}$ (103)

$h_{\inf}=\frac{1}{1+\exp((V+53)/(1))}$ (104)

where $\tau_{m}=8\text{ (ms), }\tau_{\text{h}}=1\text{ }(ms)$ modified

***Low threshold activated calcium current (Poirazi et al., 2003)***

$I_{Ca(T)}=g_{Ca(T)_{init}}*m^{2}*h*(V-E_{Ca})$ (105)

$\frac{dm}{d(t)}=\frac{m_{inf}-m(t,V)}{\tau_{m}(V)}$ (106)

$m_{inf}=\frac{1}{1+a}$ (107)

$a=alpm(V)$ (108)

$alpm(V)=exp(\frac{(1.e-3*zetam*(V-V_{halfm})*9.648e4}{(8.315*(273.16+celsius))})$ (109)

where$\tau_{m}$ = 1.5 $(ms)$ $zetam$ = -3, $V_{halfm}$ =-36 $(mV)$.

For the inactivation state h:

$\frac{dh}{dt}=\frac{(h_{inf}-h)}{\tau_{h}}$ (110)

$h_{inf}=\frac{1}{1+b}$ (111)

$b=alph(v)$ (112)

$alph=exp(\frac{1.e-3*zetah*(V-V_{halfh})*9.648e4}{(8.315*(273.16+celsius))})$ (113)

where $\tau_{h}$ = 10$(ms)$ $zetah$ = 5.2, $V_{halfh}$ = -68 $(mV)$

***The fast calcium dependent potassium current*** $E_{K_{fAHP}}$

$$I_{\left[K\right]}=g_{K}*O*(V-E_{Ca})$$

The channel has three states according to (Shao et al., 1999): active (O), non active (C) and inactivated (I).

The transition from C🡪O is calcium dependent. The transition from O🡪I is voltage dependent and is responsible for the presence of the fast afterhyperpolarization potential. The transition from I🡪C is voltage dependent and slow as well.

$$\begin{matrix} C\leftrightarrow O (K_{3}, K_{4}) \\ O\leftrightarrow I (K_{1}, 0) \\ I\leftrightarrow C (K_{2}, 0) \\ C+O+I=1 \end{matrix}$$

where K_1_, K_2_, K_4_ are described by function $\alpha_{2}(V)$and K_3_ by function $\alpha_{1}(V)$ and correspond to the transition rates between open (O), close (C) and inactivated (I) states:

$\alpha1(V)=\frac{1}{t_{\min}+\frac{1}{\frac{1}{t_{\max}-t_{\min}}+e((V-V_{half})/K)\cdot1ms^{-1}}}*1e8*([Ca]i)e2$ (114)

$\alpha_{2}(V)=\frac{1}{t_{\min}+e^{-(\frac{V-V_{half}}{K})\cdot1ms}}$ (115)

Where

|  | $t_{\min}$ (ms) | $t_{\max}$ (ms) | $V_{half}$ (mV) | K (mV) |
| --- | --- | --- | --- | --- |
| K1 | 0.1 | - | -10 | 1.0 |
| K2 | 0.1 | - | -120 | -10.0 |
| K3 | 0.001 | 1.0 | -20 | 7.0 |
| K4 | 0.01 | - | -44 | -5.0 |

***The slow calcium dependent potassium channel*** $I_{K_{sAHP}}$

$I_{K(sAHP)}=\overline{g}_{sAHP}\cdot m^{2}\cdot(V-E_{K})$ (116)

$\frac{dm(t,V)}{d(t)}=\frac{m_{inf}-m(t,V)}{\tau_{m}(V)}$ (117)

$m_{inf}=\frac{a}{(a+1)}$ (118)

$a=[Ca^{2+}]/b$ (119)

$\tau_{m}=\frac{1(ms)*1(mM)}{[Ca^{2+}]_{i}+b)}$ (120)

where b = 0.008

***Hyperpolarization Activated Current I_h_ (Poirazi et al., 2003)***

$I_{h}=\overline{g}_{h}\cdot n\cdot(V-E_{h})$ (122)

$\frac{dn}{dt}=\frac{n_{inf}-n}{\tau_{n}},\tau_{n}=1.0(ms)$ (123)

$n_{\inf}=1-\frac{1}{1+\exp(-90-v)/10}$ (124)

where, E_h_=-10mV

***Slow inactivating potassium current (Durstewitz and Seamans, 2002)***

$I_{K}=\overline{g}_{Ks}\cdot a\cdot b\cdot(V-E_{K})$ (125)

$\frac{da}{dt}=\frac{a_{inf}-a}{\tau_{a}},\tau_{m}=1.0(ms)$ (126)

$\frac{db}{dt}=\frac{b_{inf}-b}{\tau_{b}},\tau_{m}=1.0(ms)$

$$a_{\inf}=\frac{1}{1+\exp(-(v+34)/6.5)}$$

$$\tau_{a}=10$$

$$b_{\inf}=\frac{1}{1+\exp((v+65)/6.6)}$$

$\tau_{b}=3400$ (131)

***The dADP (CAN) mechanism (Destexhe, 1994)***

$I_{n(CAN)}=\overline{g}_{CAN}\cdot m^{2}\cdot(V-E_{n})$ (132)

$I_{Na}=0.7\cdot I_{n(CAN)}$ **,** based on Fowler et al, 2007, where about 70% of the dADP is Na+ current

$\frac{dm}{dt}=\frac{m_{inf}-m}{\tau_{m}}$ (133)

$m_{\inf}=\frac{alpha}{(alpha+beta)/tadj}$ (134) $\tau_{m}=\frac{1}{(alpha+beta)/tadj}$ (135)

alpha = beta *($\frac{[Ca_{i}]}{[cac]}$)^2^ (136)

where beta = 0.0001(1/ms) and cac = 0.0004 (mM)

The beta and cac values were adjusted so that the dADP was induced following more 4 spikes and had decay kinetics in the order of a few (~3sec).

**D.** Table that shows conductance and distributions of active and passive mechanisms in the various sections of the model neuron.

RS model neuron

| **Mechanisms** | **Soma** | **Axon** | **Basal**  **Dendrites** | **Apical dendrites** |
| --- | --- | --- | --- | --- |
| *Leak=1/Rm*  *(1/K Ωcm^2^)* | 3e-4 | 3e-4 | Decreases sigmoidally up to half of the somatic conductance using the following equation: 3e-4+(1.5e-4-3e-4)/ (1.0+ exp(10-<distance>/5) | Decreases sigmoidally up to half of the somatic conductance using the following equation: 3e-4 + (1.5e-4-3e-4)/ (1.0+ exp(300-<distance>/50) |
| *Na* | 0.155 | 0.31 | 0.0031 | 0.0062 |
| *Kdr* | 0.045 | 0.045 | 0.0045 | 0.000045 |
| *Nap* | 0.6e-5  (*2 for IB) | 0 | 0.6e-5 | If distance from the soma <200μm: 0.6e-5  If distance from the soma >200μm: 3e-5 *(<distance>/200) |
| *A-type* | 9e-4 | 0 | If distance from the soma <50μm: 18e-4  If distance from the soma >50μm: 45e-4 | If distance from the soma <100μm: 9e-4  If distance from the soma >100μm but <300μm: 9e-5*(300/<distance>)  If distance from the soma >300μm: 9e-5 |
| D-type | 5.28e-3 | 0 | 0 | If distance from the soma <100μm: 5.28e-3  If distance from the soma >100μm but <300μm: 5.28e-4*(300/<distance>)  If distance from the soma >300μm: 5.28e-4 |
| *N-type CC* | 0.3e-5 | 0 | If distance from the soma <50μm: 0.3e-5  If distance from the soma >50μm: 45e-4 | If distance from the soma <200μm: 0.3e-5  If distance from the soma >200μm: 0.96e-5*(<distance>/200) |
| *T-type CC* | 1e-4 | 0 | 1e-5 | If distance from the soma <200μm: 1e-4  If distance from the soma >200μm: 1e-4* (<distance>/200) |
| *CaR* | 37.5e-5 (*2 for IB) | 0 | 0 | If distance from the soma <200μm: 37.5e-5  If distance from the soma >200μm: 37.5e-5* (<distance>/200) |
| *L-type* | 3e-4 | 0 | 0 | If distance from the soma <200μm: 3e-4  If distance from the soma >200μm: 3e-4*(30/<distance>) |
| *sAHP* | 1.25e-2 | 0 | 0 | If distance from the soma <200μm: 1.25e-3  If distance from the soma >200μm: 1.25e-6 |
| *fAHP* | 3e-5 | 0 | 0 | If distance from the soma <200μm: 15e-7  If distance from the soma >200μm: 3e-8 |
| *H-current* | 9.36e-6 | 0 | 9.36e-6 | Increases sigmoidally up to 10x the somatic conductance using the following equation: 9.36e-6+(93.6e-6-9.36e-6)/(1.0+exp(300-<distance>/50) |
| *dADP current* | 1e-4 | 0 | 1e-5 | 1e-5 |
| *Calcium diffusion model* | Yes | No | Yes | Yes |

E. References

Day M, Carr DB, Ulrich S, Ilijic E, Tkatch T, Surmeier DJ (2005) Dendritic excitability of mouse frontal cortex pyramidal neurons is shaped by the interaction among HCN, Kir2, and Kleak channels. JNeurosci 25:8776-8787.

de la PE, Geijo-Barrientos E (1996) Laminar localization, morphology, and physiological properties of pyramidal neurons that have the low-threshold calcium current in the guinea-pig medial frontal cortex. JNeurosci 16:5301-5311.

Destexhe A (1994) Oscillations, complex spatiotemporal behavior, and information transport in networks of excitatory and inhibitory neurons. PHYSICALREVIEWESTATISTICALPHYSICS, PLASMAS, FLUIDS, AND RELATEDINTERDISCIPLINARYTOPICS 50:1594-1606.

Destexhe A, Sejnowski TJ (1995) G protein activation kinetics and spillover of gamma-aminobutyric acid may account for differences between inhibitory responses in the hippocampus and thalamus. Proc Natl Acad Sci U S A 92:9515-9519.

Destexhe A, Mainen ZF, Sejnowski TJ (1997) Kinetic Models of Synaptic Transmission. In: Methods in Neuronal Modeling, 2nd Edition (Koch C, Segev I, eds). Cambridge, MA: MIT Press.

Durstewitz D, Seamans JK (2002) The computational role of dopamine D1 receptors in working memory. Neural Netw 15:561-572.

Durstewitz D, Gabriel T (2007) Dynamical basis of irregular spiking in NMDA-driven prefrontal cortex neurons. Cereb Cortex 17:894-908.

Fowler MA, Sidiropoulou K, Ozkan ED, Phillips CW, Cooper DC (2007) Corticolimbic Expression of TRPC4 and TRPC5 Channels in the Rodent Brain. PLoS ONE 2:e573.

Gonzalez-Burgos G, Barrionuevo G (2001) Voltage-gated sodium channels shape subthreshold EPSPs in layer 5 pyramidal neurons from rat prefrontal cortex. J Neurophysiol 86:1671-1684.

Hagenston AM, Fitzpatrick JS, Yeckel MF (2008) MGluR-mediated calcium waves that invade the soma regulate firing in layer V medial prefrontal cortical pyramidal neurons. Cereb Cortex 18:407-423.

Haj-Dahmane S, Andrade R (1998) Ionic mechanism of the slow afterdepolarization induced by muscarinic receptor activation in rat prefrontal cortex. JNeurophysiol 80:1197-1210.

Kole MH, Hallermann S, Stuart GJ (2006) Single Ih channels in pyramidal neuron dendrites: properties, distribution, and impact on action potential output. J Neurosci 26:1677-1687.

Korngreen A, Sakmann B (2000) Voltage-gated K+ channels in layer 5 neocortical pyramidal neurones from young rats: subtypes and gradients. J Physiol 525 Pt 3:621-639.

Liebmann L, Karst H, Sidiropoulou K, van Gemert N, Meijer OC, Poirazi P, Joels M (2008) Differential effects of corticosterone on the slow afterhyperpolarization in the basolateral amygdala and CA1 region: possible role of calcium channel subunits. J Neurophysiol 99:958-968.

Lorenzon NM, Foehring RC (1992) Relationship between repetitive firing and afterhyperpolarizations in human neocortical neurons. J Neurophysiol 67:350-363.

Lorenzon NM, Foehring RC (1995) Characterization of pharmacologically identified voltage-gated calcium channel currents in acutely isolated rat neocortical neurons. I. Adult neurons. JNeurophysiol 73:1430-1442.

Nevian T, Larkum ME, Polsky A, Schiller J (2007) Properties of basal dendrites of layer 5 pyramidal neurons: a direct patch-clamp recording study. Nat Neurosci 10:206-214.

Pissadaki EK, Poirazi P (2007) Modulation of excitability in CA1 pyramidal neurons via the interplay of entorhinal cortex and CA3 inputs. Neurocomputing 70:1735-1740.

Poirazi P, Brannon T, Mel BW (2003) Online Supplement: About the Model. . Neuron 37.

Schaefer AT, Helmstaedter M, Schmitt AC, Bar-Yehuda D, Almog M, Ben-Porat H, Sakmann B, Korngreen A (2007) Dendritic voltage-gated K+ conductance gradient in pyramidal neurones of neocortical layer 5B from rats. J Physiol 579:737-752.

Shao LR, Halvorsrud R, Borg-Graham L, Storm JF (1999) The role of BK-type Ca2+-dependent K+ channels in spike broadening during repetitive firing in rat hippocampal pyramidal cells. J Physiol 521 Pt 1:135-146.

Wang H, Stradtman GG, 3rd, Wang XJ, Gao WJ (2008) A specialized NMDA receptor function in layer 5 recurrent microcircuitry of the adult rat prefrontal cortex. Proc Natl Acad Sci U S A 105:16791-16796.

Woo RS, Li XM, Tao Y, Carpenter-Hyland E, Huang YZ, Weber J, Neiswender H, Dong XP, Wu J, Gassmann M, Lai C, Xiong WC, Gao TM, Mei L (2007) Neuregulin-1 enhances depolarization-induced GABA release. Neuron 54:599-610.
